# Low SARS-CoV-2 viral load among vaccinated individuals infected with Delta B.1.617.2 and Omicron BA.1.1.529 but not with Omicron BA.1.1 and BA.2 variants

**DOI:** 10.3389/fpubh.2022.1018399

**Published:** 2022-09-20

**Authors:** Sivaprakasam T. Selvavinayagam, Yean Kong Yong, Narcisse Joseph, Kannan Hemashree, Hong Yien Tan, Ying Zhang, Manivannan Rajeshkumar, Anandhazhvar Kumaresan, Raghu Kalpana, Vasudevan Kalaivani, Ayyagari Venkata Devi Monika, Suvaiyarasan Suvaithenamudhan, Meganathan Kannan, Amudhan Murugesan, Krishnasamy Narayanasamy, Sampath Palani, Marie Larsson, Esaki M. Shankar, Sivadoss Raju

**Affiliations:** ^1^Directorate of Public Health and Preventive Medicine, Chennai, India; ^2^Laboratory Centre, Xiamen University Malaysia, Sepang, Malaysia; ^3^Faculty of Medicine and Health Sciences, Universiti Putra Malaysia, Serdang, Malaysia; ^4^School of Traditional Chinese Medicine, Xiamen University Malaysia, Sepang, Malaysia; ^5^Chemical Engineering, Xiamen University Malaysia, Sepang, Malaysia; ^6^Infection Biology, Department of Life Sciences, Central University of Tamil Nadu, Thiruvarur, India; ^7^Blood and Vascular Biology, Department of Life Sciences, Central University of Tamil Nadu, Thiruvarur, India; ^8^Department of Microbiology, The Government Theni Medical College and Hospital, Theni, India; ^9^Government Corona Hospital, Guindy, Chennai, India; ^10^Molecular Medicine and Virology, Department of Biomedicine and Clinical Sciences, Linkoping University, Linköping, Sweden

**Keywords:** AZD1222, BBV152, COVID-19 severity, Omicron BA.2, phylogeny

## Abstract

The rapid spread of SARS-CoV-2 variants in the global population is indicative of the development of selective advantages in emerging virus strains. Here, we performed a case-control investigation of the clinical and demographic characteristics, clinical history, and virological markers to predict disease progression in hospitalized adults for COVID-19 between December 2021 and January 2022 in Chennai, India. COVID-19 diagnosis was made by a commercial TaqPath COVID-19 RT-PCR, and WGS was performed with the Ion Torrent Next Generation Sequencing System. High-quality (<5% of N) complete sequences of 73 Omicron B.1.1.529 variants were randomly selected for phylogenetic analysis. SARS-CoV-2 viral load, number of comorbidities, and severe disease presentation were independently associated with a shorter time-to-death. Strikingly, this was observed among individuals infected with Omicron BA.2 but not among those with the BA.1.1.529, BA.1.1, or the Delta B.1.617.2 variants. Phylogenetic analysis revealed severe cases predominantly clustering under the BA.2 lineage. Sequence analyses showed 30 mutation sites in BA.1.1.529 and 33 in BA.1.1. The mutations unique to BA.2 were T19I, L24S, P25del, P26del, A27S, V213G, T376A, D405N and R408S. Low SARS-CoV-2 viral load among vaccinated individuals infected with Delta B.1.617.2 and the Omicron BA.1.1.529 variant but not with Omicron BA.1.1 or BA.2 suggests that the newer strains are largely immune escape variants. The number of vaccine doses received was independently associated with increased odds of developing asymptomatic disease or recovery. We propose that the novel mutations reported herein could likely bear a significant impact on the clinical characteristics, disease progression, and epidemiological aspects of COVID-19. Surging rates of mutations and the emergence of eclectic variants of SARS-CoV-2 appear to impact disease dynamics.

## Introduction

The COVID-19 pandemic has resulted in an unprecedented global emergency and has claimed more than 6.51 million deaths by September 2022 (https://covid19.who.int/). COVID-19 has had a devastating impact on global health and economy. While antiviral agents against the severe acute respiratory syndrome coronavirus 2 (SARS-CoV-2), the virus causing COVID-19, are yet to become widely available (1), vaccines and public health interventions recommended by the World Health Organization (WHO) remain the most promising approach against the global catastrophe (2). Being a new virus strain encountered by the human host, the SARS-CoV-2 virus appears to undergo a series of mutations to adapt itself into the human population that likely could alter the disease spectrum, presentation and dynamics in the coming years (3). Eclectic variants of SARS-CoV-2 are increasingly evolving globally throughout the pandemic.

A SARS-CoV-2 variant is defined as owning one or more genetic mutations that distinguishes it from other virus variants. During its evolution the virus can either become more infectious/transmissible, or more efficient to evade the host's defense (4–6). The wild-type SARS-CoV-2 has evolved into several variants and sub-variants, some of which were identified as variants of concern (VoC) by the WHO such as the B.1.1.7 and Q pango (the Alpha variant), the B.1.351 and descendent pango (the beta variant), the P.1 and descendent pango (the Gamma variant). This was followed by the much alarming Delta variant (B.1.617.2 and AY) identified in Maharashtra, India during a pandemic wave that swept the country in mid-2021 (7). The Omicron B.1.1.529 variant possesses a fitness advantage over the Delta B.1.617.2 variant and continues to evolve actively (4). The mutation-laden lineages of SARS-CoV-2 are routinely monitored via epidemiological, sequence-based surveillance, and laboratory investigations.

The Omicron variant appears to harbor several genetic mutations in the spike protein, particularly in the S1 and S2 regions, more specifically involving the receptor-binding domain (RBD), which binds the ACE2 protein expressed on a broad array of host cells. Importantly, the Omicron variant has branched out into the B.1.1.529, BA.1, BA.1.1, BA.2, BA.3, BA.4, BA.5 and BA.7 sub-variants (8). Of note, the BA.1 represents a dominant mutant that reportedly escapes from neutralizing antibodies induced by vaccination (9, 10). The surge in infections by BA.2 suggests that the variant harbors a selective advantage over BA.1. Despite reports that BA.1 and BA.2 share several mutations in common, each of the variants possess unique mutations (8). Having said that virus variants can alter the disease presentation and pathogenesis attributes, additional studies are needed to determine whether the SARS-CoV-2 viral load (VL) within the respiratory tract may predict disease characteristics. Here, we performed a case-control study of the clinico-demographic features, clinical history, and virological markers to predict COVID-19 progression in hospitalized adults. We also performed phylogenetic analyses and conducted a detailed investigation on sequence variations to determine mutations in the spike protein of the virus variants. The primary objective of the current investigation was to underpin the diverse factors (including vaccine doses) associated with COVID-19 progression.

## Materials and methods

### Study population

The case-control study recruited 287 hospitalized adults for COVID-19-related illness at the Government Corona Hospital, Chennai, India from December 2021 until January 2022. The inclusion criteria were that the participants needed to be >18 years of age, and there were no exclusion criteria. Nasopharyngeal swabs were collected from participants for routine COVID-19 diagnosis. The standard demographic details such as age, gender, vaccination status, type of vaccine received, history of SARS-CoV-2 infection, underlying comorbidities, COVID-19 symptoms and signs, and treatment outcomes were obtained from medical records. The study procedures and/or protocols were reviewed and approved by the Human Ethics Committee of the Madras Medical College (MMC) (EC No. 03092021). All patients/participants provided their written informed consent to participate in the investigation.

### Clinical classification of COVID-19 severity

The clinical classification of the study participants was based on the *Clinical Guidance for Management of Adult COVID-19 Patients* by the Ministry of Health and Family Welfare, Government of India (January 2022). Accordingly, individuals were defined as having mild COVID-19 if they had upper respiratory tract symptoms and/or fever without shortness of breath or hypoxia. Moderate disease cases reported any one of the following manifestations viz., respiratory rate ≥24/min, breathlessness or a SPO_2_ of 90% to ≤93% on room air that warranted hospitalization. Participants were defined as having severe/critical disease if they had one or more of the following manifestations of COVID-19: Respiratory rate >30/min, or breathlessness a SPO_2_ of <90% on room air, which required admission in HDU/ICU (for close treatment and monitoring).

### Detection and identification of SARS-CoV-2 variants

Diagnosis of COVID-19 was made based on clinical and laboratory diagnoses; the former based on Universal Clinical Criteria 2021 defined by the Centers for Disease Control and Prevention (CDC), Atlanta, USA (https://ndc.services.cdc.gov/case-definitions/coronavirus-disease-2019–2021/), and the later confirmed by a commercial TaqPath^TM^ SARS-CoV-2 RT-PCR (Applied Biosystems, Thermo Fisher Scientific, Pleasanton, CA) for the qualitative detection of nucleotides/genome sequences of SARS-CoV-2.

### SARS-CoV-2 viral RNA extraction

All samples selected for sequencing had RNA freshly extracted from the primary sample source independent of the material extracted for the initial SARS-CoV-2 RT-PCR. RNA extraction was carried out using a commercial MagMAX^TM^ Viral/Pathogen II Nucleic Acid isolation kit (Applied Biosystems, Thermo Fisher Scientific, USA) as per the manufacturer's instructions.

### Whole genome sequencing

Copy DNA was prepared using the SuperScript VILO cDNA synthesis kit (Invitrogen, Thermo Fisher Scientific, USA). SARS-CoV-2 library was prepared using 10μl of cDNA by an Ion AmpliSeq kit for Chef DL8 (Thermo Fisher Scientific), and was adjusted to 75 pM before loading onto an Ion Chef instrument for emulsion PCR, enrichment, and subsequently onto an Ion 540 chip. WGS was performed using the Ion Torrent NGS System using an Ion GenStudio S5 Plus System (Thermo Fisher Scientific, USA). Raw data were analyzed using the Torrent Suite software v5.12.0, and the NGS QC Toolkit v 2.3.3 was employed to ward-off low-quality and short reads. Variant Caller v5.10.1.19 was used to detect variants, compared to the Wuhan-Hu-1 genome (GenBank accession number MN908947.3), and the consensus sequence was developed using IRMAreport v1.3.0.2. The annotation was performed using COVID19AnnotateSnpEff v1.3.0.2, a plugin specifically developed for SARS-CoV-2 to predict the effect of base substitution.

### Phylogenetic analysis

High-quality (<5% of N) and complete sequences of the Omicron sub-lineages (*n* = 73) were included for the phylogenetic analyses (sequence details available in Supplementary Table 1) (based on the availability of their complete sequences). The FASTA files of the genomes were aligned using a multiple sequence alignment program MAFFT (https://mafft.cbrc.jp/alignment/server). The phylogenetic tree was constructed using Molecular Evolutionary Genetics Analysis tool (MEGA 11) (11). Maximum likelihood algorithm with Kimura 2 model were employed and the coronavirus isolate *Wuhan-Hu-1* were used to root the tree (12). A circular view of the phylogenetic supertree was designed and constructed using the iTOL server v6.0 (13).

Sequence analyses were performed on the four lineages to determine the spike protein mutations using SARS-CoV-2 (hCoV-19) Lineage Comparison tool (https://outbreak.info/compare-lineages) (14, 15). The sequence analysis was conducted with 170,225 B.1.617.2 sequences, 493,145 BA.1.1.529 sequences, 936,505 BA.1.1 sequences and 1,079,725 BA.2 sequences from GISAID Initiative as of 20 July 2022.

### Statistical analyses

Comparison of categorical variables was tested using the Chi-Square test, whereas continuous variables (e.g., age) were compared using the unpaired *t-*test. Potential risk factors of disease severity such as patient demography, vaccination status, vaccine and number of doses received, comorbidities and viral variants, were evaluated using univariate and multivariate binary logistic regression (16). The odds ratio (OR) and 95% confidence interval (CI) were estimated. Statistical analyses were performed using GraphPad PRISM, ver5.02 (GraphPad, San Diego, CA). Binary regression was performed using SPSS, ver20 (IBM, Armonk, NY), two-tailed *P* < 0.05 was considered as statistical significance for all the tests performed, and *P* values <0.05, <0.01, <0.001, <0.0001 were marked as ^*^, ^**^, ^***^ and ^****^, respectively.

## Results

### Clinico-demographic and patient characteristics

A total of 287 SARS-CoV-2-infected patients who were hospitalized for COVID-19 were recruited to the current study. The median age was 73 years (IQR = 64–80 years) and a vast majority were male (68.6%). Among these, only less than half (*n* = 137; 47.7%) were vaccinated. In India, two vaccines were initially approved for administration to the public, one the replication-deficient chimpanzee adenoviral vector-based AZD1222 (ChAdOx1) (manufactured by the Serum Institute of India, Pune), and the other, a whole-virion inactivated BBV152 vaccine (marketed by the Bharat Biotech International Limited, Hyderabad, in collaboration with the Indian Council of Medical Research, New Delhi) (17). In this cohort, 62.8% patients received AZD1222 and 37.2% received BBV152. These vaccinated individuals had a wide range of symptoms ranging between asymptomatic, mild to moderate and severe, and their percentages were 6.3, 30.3, 47.4, and 16%, respectively. Variants identification showed that 17.4% were Omicron BA.1, 57.5% were Omicron BA.1.1, 21.9% were Omicron BA.2 and only 3% were Delta B.1.617.2 variants. The median SARS-CoV-2 viral load was 5.8 log_10_ copies/ml (IQR = 4.5–6.4 log_10_ copies/ml). Almost all, i.e., 99.3% patients required hospitalization and among them 42.5% required HDU/ICU and other medical supports, including oxygen support (11.8%) and mechanical ventilation (11.8%). Two hundred and thirty-seven (82.6%) hospitalized patients succumbed to COVID-19 during the course of treatment (Table 1) likely due to various underlying/predisposing factors, including comorbidities (if any).

**Table 1:** Patient clinico-demographic characteristics to study the effect of COVID-19 vaccination on Delta and Omicron nasopharyngeal viral loads.

**Characteristic**	**Total (*N =* 287)**	**SARS-CoV-2 variants**				
		**Delta B.1.617.2**	**Omicron BA.1**	**Omicron BA.1.1**	**Omicron BA.2**	***P*-value^†^**
Number, *n* (%)	287	9 (3.1)	50 (17.4)	165 (57.5)	63 (21.9)	–
Age; years, median (IQR)	73 (64–80)	75 (63–80.5)	71 (59.5–76)	73 (65–81)	72 (65–80)	0.353
Gender; male, *n* (%)	179 (68.6)	4 (44.4)	36 (72)	110 (66.7)	47 (74.6)	0.256
Viral load, Log_10_ copies ml^−1^	5.8 (4.5–6.4)	4.5 (4.2–5.5)	4.7 (3.6–5.8)	6.1 (4.8–6.7)	5.8 (4.8–6.7)	<0.0001^a^
Comorbidities, *n* (%)	225 (78.4)	8 (88.9)	35 (70)	132 (80)	50 (79.4)	0.399
Vaccination; Yes, *n* (%)	137 (47.7)	3 (33.3)	29 (58)	71 (43)	34 (54)	0.151
**Type of vaccine;** ***n*** **(%)**
AZD1222	86 (30)	2 (22.2)	16 (32)	49 (29.7)	19 (30.2)	0.348
BBV152	51 (17.1)	1 (11.1)	12 (24)	24 (14.5)	14 (22.2)	0.255
**No. of vaccine doses;** ***n*** **(%)**
1 dose	24 (8.4)	1 (11.1)	5 (10)	13 (7.9)	5 (7.9)	0.524
2 doses	113 (39.4)	2 (22.2)	23 (46)	58 (35.2)	30 (47.6)	0.543
**Severity;** ***n*** **(%)**
Asymptomatic	18 (6.3)	1 (11.1)	4 (8)	9 (5.5)	4 (6.3)	0.849
Mild	87 (30.3)	2 (22.2)	19 (38)	51 (30.9)	15 (23.8)	0.397
Moderate	136 (47.4)	5 (55.6)	22 (4.4)	83 (50.3)	26 (41.3)	0.577
Severe	46 (16)	1 (11.1)	5 (10)	22 (13.3)	18 (28.6)	0.021^*^
**Medical support;** ***n*** **(%)**
Hospitalization	285 (99.3)	9 (100)	50 (100)	164 (99.4)	62 (98.4)	0.764
HDU/ICU	122 (42.5)	4 (44.4)	25 (50)	76 (46.1)	15 (23.8)	0.021^*^
O_2_ support	120 (41.8)	4 (44.4)	25 (50)	77 (46.7)	16 (25.4)	0.021^*^
Ventilator	34 (11.8)	2 (22.2)	9 (18)	20 (12.1)	3 (4.8)	0.123
Death; *n* (%)	237 (82.6)	8 (88.9)	31 (62)	140 (84.8)	58 (92.1)	<0.0001^****^
Day to death; day, median (IQR)	3 (1–6)	5.5 (2.5–15.3)	3 (2–6)	3 (1–6)	4 (2–6.3)	0.163

All data reported as numbers (n) and percentages (%) unless specified. HDU, High dependency unit; IQR, interquartile range; ICU, intensive care unit; O_2_, Oxygen. ^*^, ^**^, ^***^, ^****^ represent P <0.05, <0.01, <0.001 and <0.0001, respectively.

† represent comparisons was made between different variants of SARS-CoV-2.

**Figure 1:**
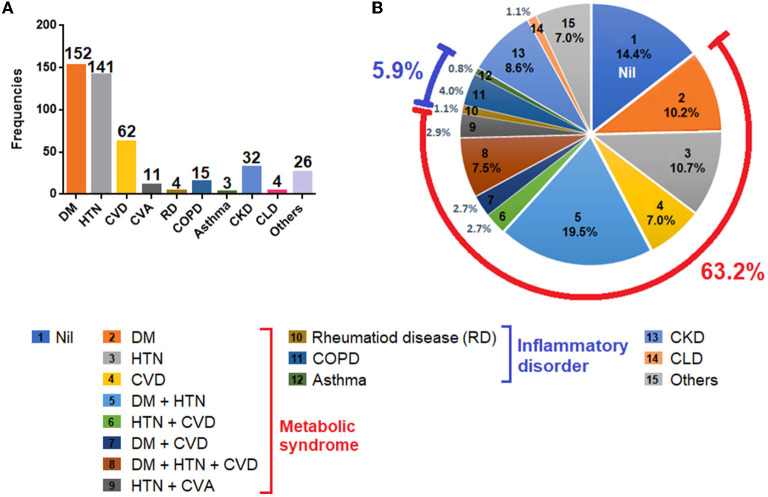
Comorbidities observed in the patient cohort. **(A)** Frequencies of comorbidities reported in the cohort. **(B)** Percentages of each comorbidities and in combinations. Footnotes: Nil, patients with no comorbid conditions; DM, diabetes mellitus; HTN, hypertension; CVD, cardiovascular disease (includes coronary artery disease, ischemic heart disease); CVA, cerebrovascular accident (stroke); RD, rheumatic disease (includes rheumatic arthritis, rheumatic heart disease); COPD, chronic obstructive pulmonary disease; CKD, chronic kidney disease; CLD, chronic liver disease; others, includes hypothyroidism, (*n* = 7), malignancies (*n* = 4), pulmonary tuberculosis (*n* = 4), Parkinson's disease (*n* = 3), seizure disorder (*n* = 2), anemia (*n* = 3); psychiatric disorders (*n* = 2), and biliary atresia (*n* = 1).

Besides the high fatality (i.e., 82.6%), the cohort also identified multiple comorbidities in a vast majority of the deceased cases. The notable comorbid conditions in the current cohort included diabetes mellitus (DM), hypertension (HTN), cardiovascular disease (CVD), rheumatic disease (RD), chronic obstructive pulmonary disease (COPD), asthma, CKD, and CLD. Among all comorbidities, DM (53%), HTN (49%) and CVD (21.6%) were the most common (Figure 1A). As DM, HTN and CVD were often clustered together in different combinations, and because these conditions are considered as ingredients of metabolic syndrome (18), the cohort identified a large proportion of patients (63.2%) having at least one of these conditions. The cohort also reported inflammatory disease (5.9%), CKD (8.6%) and CLD (1.1%) (Figure 1B).

### Sequence analyses revealed 30 mutation sites in BA.1.1.529 and 33 in BA.1.1

**Figure 2:**
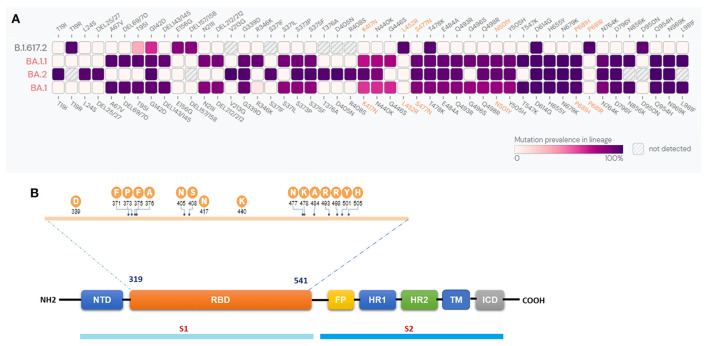
**(A)** Comparison of spike protein mutations of the Omicron BA.11.529, BA.1.1, BA.2, and Delta B.1.617.2 lineages. The color indicates the prevalence of S protein mutations from available sequences from GISAID Initiative as of 20th July 2022. **(B)** SARS-CoV-2 spike monomer genome arrangement. N-terminal domain (NTD), receptor binding domain (RBD), fusion peptide (FP), heptad repeat (HR1 and 2), trans-membrane region (TM), intracellular domain (ICD).

Sequence analysis was performed to identify the mutations in the spike regions of the BA.1, BA.1.1, BA.2, and B.1.617.2 variants. Our results revealed the presence of 30 mutation sites in BA.1 and 33 in BA.1.1. Further, BA.2 reported 29, and B.1.617.2 revealed 8 mutation sites (Figure 2A). The common mutations present across all the four variants were G142D, T478K, and D614G, whereas mutations G339D, S373P, S375F, K417N, N440K, S477N, E484A, Q493R, Q498R, N501Y, Y505H, H655Y, N679K, P681H, N764K, D796Y, Q954H, and N969K were commonly found in all the three BA.1, BA.1.1, BA.2 Omicron variants. The major mutation del69/70 commonly reported in BA.1.1.529 and BA.1.1 (19) was not present in the BA.2 as well as the Delta variant B.1.617.2. The mutations unique to BA.2 were T19I, L24S, P25del, P26del, A27S, V213G, T376A, D405N and R408S. Figure 2B illustrates the SARS-CoV-2 spike monomer genome arrangement. The sequence information of all the 73 omicron variants is provided in Supplementary Table 1.

### SARS-CoV-2 viral load was associated with increased risk for development of severe COVID-19 pneumonia and death

Because a substantial proportion of individuals developed moderate and severe COVID-19 symptoms, many of them succumbed to the disease despite the extension of extensive medical supports such as HDU/ICU, ventilator and oxygen support. Hence, we sought to investigate the factors associated with development of critical/severe COVID-19 and death. The association between disease severity, medical support required, and treatment outcomes with demographic parameters such as age, gender, SARS-CoV-2 viral load, vaccination status, type and number of vaccine doses received, comorbidities and SARS-CoV-2 variants were first assessed univariately using a binary regression model.

**Figure 3:**
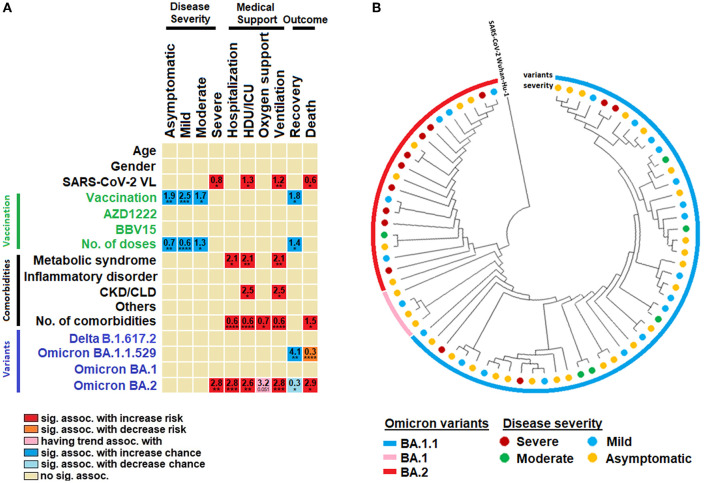
Factors associated with disease severity. **(A)** Univariate binary regression analysis of factors associated with disease severity, use of medical supports and disease prognosis. **(B)** A circular view of the phylogenetic tree representing the origin of omicron variants. The variants and their disease severity are grouped and classified in their respective colors. Sig. assoc., significant association; ICU, intensive care unit; CKD, chronic kidney disease; CLD, chronic liver disease. *, **, ***, **** represent *P* < 0.05, <0.01, <0.001 and <0.0001, respectively.

Our analysis showed that the SARS-CoV-2 viral load was significantly associated with increased risk for the onset of severe disease, HDU/ICU admission, use of mechanical ventilation and death. Every increase of viral load by 1 log was associated with increased risk for severe disease, HDU/ICU, use of ventilator and death by 0.79, 1.27, 1.22, and 0.56, respectively (Table 2). Vaccination was significantly associated with increased chances of asymptomatic/mild/moderate symptoms as well as recovery. Number of vaccine doses administered were also significantly associated with increased odds for development of asymptomatic/mild/moderate disease as well as recovery; where every single dose of vaccine received was associated with increase chances of developing asymptomatic, mild, moderate COVID-19 and recovery by 0.6, 07, 1.3, and 1.43, respectively (Figure 3A). There was no significant difference between use of the two different types of vaccines (i.e., AZD1222 and BBV15) vis-à-vis their association with disease severity and survival rate. Metabolic syndrome (DM, hypertension, CVD and other comorbidities mentioned herein) and CKD/CLD were the two main categories of comorbid conditions associated with COVID-19 sequelae/complications.

**Table 2:** Multi-variate analysis of factors associated with disease severity, medical support required and treatment outcomes.

**Outcome**	**Variable**	**Coeff. (95% CI)**	***P-*value**
Asymptomatic and mild disease	SARS-CoV-2 VL	1.05 (0.89, 1.25)	0.563
	No. of vaccine doses	0.69 (0.53, 0.89)	0.004^**^
	No. of comorbidities	1.06 (0.85, 1.32)	0.594
	Omicron BA.2	0.64 (0.35, 1.19)	0.163
Severe disease	SARS-CoV-2 VL	1.28 (1.08, 1.63)	0.043^*^
	No. of vaccine doses	0.82 (0.58, 1.16)	0.259
	No. of comorbidities	0.97 (0.73, 1.29)	0.827
	Omicron BA.2	0.34 (0.17, 0.68)	0.002^**^
Oxygen support	SARS-CoV-2 VL	1.25 (1.04, 1.49)	0.015^*^
	No. of vaccine doses	1.03 (0.79, 1.34)	0.856
	No. of comorbidities	0.61 (0.48, 0.77)	<0.0001^****^
	Omicron BA.2	3.05 (1.57, 5.93)	0.001^***^
HDU/ICU	SARS-CoV-2 VL	1.25 (1.05, 1.49)	0.014^*^
	No. of vaccine doses	1.07 (0.82, 1.39)	0.639
	No. of comorbidities	0.6 (0.47, 0.76)	<0.0001^****^
	Omicron BA.2	2.87 (1.49, 5.52)	0.002^**^
Ventilator	SARS-CoV-2 VL	1.26 (0.89, 1.50)	0.255
	No. of vaccine doses	1.17 (0.80, 1.72)	0.410
	No. of comorbidities	0.71 (0.52, 1.01)	0.054^†^
	Omicron BA.2	3.35 (0.98, 11.5)	0.054^†^
Recovery	SARS-CoV-2 VL	0.93 (0.75, 1.16)	0.534
	No. of vaccine doses	1.35 (0.97, 1.88)	0.052^†^
	1 dose	1.00 (ref)	–
	2 doses	1.83 (0.94, 3.56)	0.054^†^
Death	SARS-CoV-2 VL	1.07 (0.86, 1.33)	0.534
	No. of vaccine doses	0.74 (0.53, 1.03)	0.052^†^
	No. of comorbidities	1.47 (1.09, 1.98)	0.012^*^
	Omicron BA.2	3.6 (1.14, 8.18)	0.026^*^

All data reported as median of coefficients, 95% confident intervals; p-values. ^**^, ^***^, ^****^ represent P < 0.05, <0.01, <0.001 and <0.0001, respectively.

† Having a trend of association, but not statistically significant. VL, viral load (nasopharyngeal).

Every addition of one comorbid condition was associated with increased risk of hospitalization, HDU/ICU admission, requirement of oxygen support, ventilation and death by an odds of 0.62, 0.61, 0.7, 0.6, and 1.45, respectively (Figure 3A). For SARS-CoV-2 variants, Omicron BA.1 appeared to cause less severe manifestations, whereas infection by BA.1 was associated with four-fold increased odds for recovery as compared to others. Omicron BA.2 was more pathogenic and was significantly associated with increased risk for development of severe disease, hospitalization, HDU/ICU admission, oxygen support, ventilation. and death by 2.8, 2.8, 2.6, 2.8, and 2.9 odds, respectively (Figure 3A).

### Severe COVID-19 cases were predominantly clustered under Omicron BA.2 variant

Our phylogenetic analysis identified that the severe COVID-19 cases were predominantly clustered under the Omicron BA.2 variant (Figure 3B). The phylogenetic tree was grouped and separated into one major and three sub-clades. Overall, 73 Omicron variants against reference strain *Wuhan-Hu-1* were selected for the analysis (Figure 3B). Of these, 48 viral isolates belonged to BA.1.1, four to BA.1, and 21 to BA.2 variants. The reference isolates *Wuhan-Hu-1* were considered as out-groups. Our analysis indicated that 13 strains were responsible for severe COVID-19, and 29 attributed to asymptomatic manifestations. Moreover, six moderate COVID-19-causing isolates were found among the BA.1.1 and BA.2 Omicron variants. Our findings also reveal that BA.1.1 and BA.2 were relatively more virulent than the others. Interestingly, we also found that BA.1.1.529 strain did not have any severe COVID-19 causing trait. Together, our phylogenetic analysis suggests that Omicron BA.1.1 and BA.2 variants caused more severe disease.

### The number of vaccine doses received was independently associated with only asymptomatic or mild COVID-19

In order to assess the independent influence of these factors to disease severity, we performed a multivariate analysis using linear regression controlling for clinico-demographic parameters that were previously shown to be associated with disease severity in the univariate analysis. Our multivariate model showed that the virology factors i.e., SARS-CoV-2 viral load and being infected by Omicron BA.2 were independently associated with severe disease. The model showed that every increase of SARS-CoV-2 viral load by 1 log was associated with increased risk of developing severe disease by 1.28 odds, (95% CI = 1.08–1.63; *P* = 0.043). While being infected by Omicron BA.2 variant was independently associated with increased risk of developing severe disease (0.34; 95% CI = 0.17–0.68; *P* = 0.002), the same factors were also independently associated with the requirement of oxygen support [SARS-CoV-2 viral load (1.25: 95% CI = 1.04–1.49; *P* = 0.015) Omicron BA.2 (3.05; 95% CI = 1.57–5.93; *P* = 0.001)] and admission in HDU/ICU. [SARS-CoV-2 viral load (1.25: 95% CI = 1.05–1.49; *P* = 0.014), Omicron BA.2 (2.87: 95% CI = 1.49–5.52; *P* = 0.002)]. Furthermore, factors such as comorbidities can influence medical support required for COVID-19 patients.

Our multivariate model showed that the number of underlying comorbid conditions was independently associated with increased odds for requirement of oxygen support and admission in HDU/ICU. The multivariate model showed that with every increase of one comorbid condition was significantly associated with increased odds for requirement of oxygen support (0.61: 95% CI = 0.48–0.77; *P* < 0.0001) and admission in HDU/ICU (0.6: 95% CI = 0.47–0.76; *P* < 0.0001), respectively. The number of comorbidities [1.47 (95% CI = 1.09–1.98; *P* = 0.012) and an infection with the Omicron BA.2 variant (3.6: 95% CI = 1.14–8.18; *P* = 0.026)] were the only two factors that were independently associated with death. Nonetheless, the model also showed that the number of vaccine doses received was independently associated with increased chances for developing asymptomatic and mild disease by an odds of (0.69: 95% CI = 0.53–0.89; *P* < 0.004) as well as having a trend toward recovery (Table 2).

### Omicron BA.2 and disease severity were associated with shorter time-to-death

**Figure 4:**
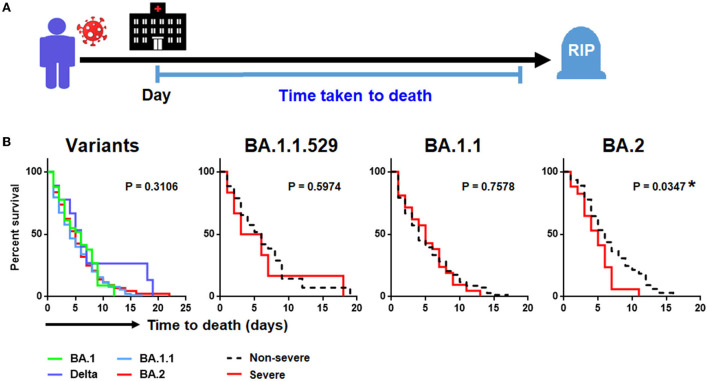
Kaplan-Meier analysis of SARS-CoV-2 infection. **(A)** Study design of time-to-death analysis. **(B)**. Factors associated with time-to-death in SARS-CoV-2 infection involving different SARS-CoV-2 variants.

Of the 237 patients (82.6%) who succumbed to COVID-19 pneumonia, there was no significant difference in time-to-death when comparing between the four SARS-CoV-2 variants (Table 1; Figure 4B). Nonetheless, by performing survival analysis, we found that those individuals who had been infected with Omicron BA.2 and those presenting with a severe disease were significantly associated with shorter time-to-death (*P* = 0.035) (Figure 4B). By using a multivariate Cox proportional hazards regression analysis controlling for covariate that previously showed to be significantly associated with death, we found that the SARS-CoV-2 viral load (0.78: 95% CI = 0.04–1.52; *P* = 0.041), number of comorbidities (0.831: 95% CI = 0.02–1.65; *P* = 0.046), and development of severe COVID-19 (3.37: 95% CI = 1.37–5.36) were independently associated with shorter time-to-death. Such association was only seen among patients infected with Omicron BA.2 but not among those infected with other variants (Table 3).

**Table 3:** Multi-variate analysis of factors associated with time-to-death.

**Variants**	**Variable**	**Coeff. (95% CI)**	***P-*value**
Delta B.1.617.2	SARS-CoV-2 VL	3.52 (−10.37, 17.42)	0.520
	No. of comorbidities	−1.11 (−6.78, 4.56)	0.616
	Severe disease	−1.44 (−52.51, 49.63)	0.941
Omicron BA.1.1.529	SARS-CoV-2 VL	3.52 (−0.59, 0.96)	0.627
	No. of comorbidities	0.03 (−1.04, 1.10)	0.950
	Severe disease	−1.44 (−52.51, 49.63)	0.941
Omicron BA.1.1	SARS-CoV-2 VL	0.26 (−0.38, 0.91)	0.424
	No. of comorbidities	0.24 (−0.47, 0.95)	0.499
	Severe disease	0.66 (−1.73, 3.04)	0.587
Omicron BA.2	SARS-CoV-2 VL	0.78 (0.04, 1.52)	0.041^*^
	No. of comorbidities	0.831 (0.02, 1.65)	0.046^*^
	Severe disease	3.37 (1.37, 5.36)	0.001^**^

^*^, ^**^, represent P < 0.05, <0.01 respectively.

### Individuals infected with Omicron BA.1.1 and BA.2 had a higher nasopharyngeal viral load

**Figure 5:**
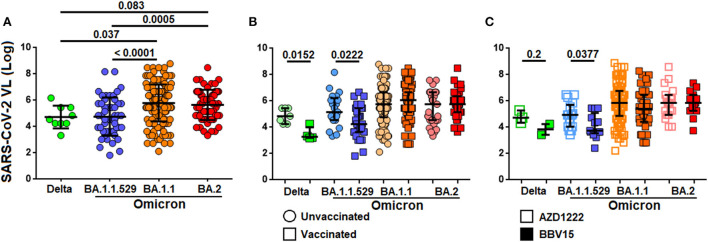
Distribution of SARS-CoV-2 viral load in different virus variants. **(A)** Levels of viral load between different SARS-CoV-2 variants. **(B)** Levels of viral load among vaccinated and non-vaccinated individuals infected by different variants of SARS-CoV-2. **(C)** Levels of viral load in individuals administered with ADZ1222 or BBV152 vaccines, and infected with different SARS-CoV-2 variants.

Given that SARS-CoV-2 viral load was consistently associated with development of more severe COVID-19, higher risk to death and shorter time to death, we investigated the levels of SARS-CoV-2 viral load in relation to the Omicron variants. We found that the viral load in the nasopharyngeal cavity was higher in patients infected with Omicron BA.1.1 and BA.2 variants as compared to those infected with the Delta B.1.617.2 and Omicron BA.1 variants by ~1.3 fold (Figure 5A). The viral load was generally low among vaccinated individuals as compared to the non-vaccinated infected with the Delta B.1.617.2 (*P* = 0.0152) and Omicron BA.1 (*P* = 0.0222) variants. However, such difference was not observed among individuals infected with the BA.1.1 and BA.2 variants (Figure 5B). When comparing the viral load between those who received the ADZ1222 and BBV152, we found that the viral load was generally lower in those who had received the BBV152 compared to those who had received the AZD1222 infected with the Delta B.1.617.2 (*P* = 0.2) and the BA.1 (*P* = 0.038) variants. Notwithstanding the median viral load in those who had received the BBV152 was seemingly low compared to those who had received the AZD1222 vaccine; due to the small sample size for Delta B.1.617.2 such association was not statistically significant. Similarly, significant difference was also not observed among patients infected with the newer Omicron variants (Figure 5C).

## Discussion

Emergence of a new virus strain is complemented with challenges such as viral immune evasion, increased transmissibility and pathogenicity/virulence, the often unsurmountable concerns associated with disease prevention and control (20). Ever since its first global report from Botswana on November 2, 2021 (21), and since December 2021, the Omicron variant has emerged as the predominant circulating strain in India causing increased rates of hospitalization and mortality. It is believed that the Omicron variant evolved in countries with poor vaccination roll-outs and among the global immunocompromised population (22). Sequence alterations in Omicron appears to increase transmissibility, drug resistance, and render escape from infection- or vaccine-induced immune responses (23). It is also evident that the Omicron transmissibility is relatively robust as compared to outbreaks caused by older SARS-CoV-2 variants (24). The current study explored whether the nasopharyngeal SARS-CoV-2 viral load could predict disease outcome. We found that the Omicron variant was significantly associated with increased risk for development of severe disease and mortality. Besides, the SARS-CoV-2 viral load as well as underlying comorbid conditions were independently associated with development of severe COVID-19, increased risk of admission in the HDU/ICU, requirement of oxygen support and death. The same risk factors also independently predicted the time-to-death when infected with the Omicron BA.2 variant. Our study also found that the viral loads were generally high among patients infected with newer variants of SARS-CoV-2, i.e. Omicron BA.1.1 and BA.2 as compared to the older circulating strains, viz., the Delta B.1.617.2 and Omicron BA.1. The viral loads were also generally lower among vaccinated individuals as well as those who had received the BBV152 but infected with the relatively older Delta B.1.617.2 and Omicron BA.1 variants. Further, our study also showed that COVID-19 vaccination was associated development of asymptomatic or mild disease. Our study supports a recent finding that described that the risk of severe outcomes following SARS-CoV-2 infection was substantially lower for the Omicron than the Delta variant largely due to the global roll-out of vaccination (25).

Ever since the emergence of the Omicron variant was reported by the WHO in November 2021 (GISAID sequence accession ID: EPI_ISL_8182767) (26), the variant has been rapidly spreading across continents leading to high morbidity rates. After the report of the first Omicron case in December 2021, the sequences were made public and thenceforth the variant has become the predominant strain in India accounting for considerable rates of morbidity. Furthermore, the identification of Omicron complements the recent surge in the number of cases in India albeit reports of relatively lesser rates of hospitalizations (27). In view of the prevailing situation, we studied the clinico-demographic characteristics, clinical history, and virological markers to predict the likelihood of an individual to develop a more severe form of COVID-19. This is seemingly critical and will a permit patient triage to render improved supportive care for the community.

A slew of studies suggests that the Omicron variant harboring a broad array of mutations resulting from 37 amino acid substitutions in the spike protein, including 15 in the RBD, displays substantial degree of escape from neutralizing antibodies induced by vaccination (9, 10, 28, 29). One study showed that over 85% of the neutralizing antibodies were successfully evaded by the Omicron variant, especially those targeting the epitopes overlapping the ACE2-binding motif, due to presence of mutations such as K417N, G446S, E484A, and Q493R (30). This is consistent with our observation where the SARS-CoV-2 viral load was lower among vaccinated individuals infected with the Delta B.1.617.2 and the Omicron BA.1 variant but not with the Omicron BA.1.1 and A.2 variants indicating that the newer variants appear to evade the immune responses induced originally by the administration of a vaccine that was developed based on the ancestral wild-type virus.

Several unique mutations have been reported among the subtypes of Omicron variants, such as BA.2 (10 mutations) and BA.1 (18 mutations) (31, 32). These Omicron subtypes have recently emerged as variants of concern (VoC) accumulating high numbers of mutations and immune evasion potential, primarily from vaccination. Variants also enforce changes in amino acid sequences, which would render them resistant to antiviral drugs as well as vaccine failure (33–37). There is compelling evidence that the emergence of variants with increased rates of mutations enhance virulence and transmissibility (38, 39). Our sequence analysis suggests the presence of 30 mutation sites in BA.1.1.529 and 33 in BA.1.1 spike regions. G142D, T478K, D614G were the common mutations present in all the four lineages, while the G339D, S373P, S375F, K417N, N440K, S477N, E484A, Q493R, Q498R, N501Y, Y505H, H655Y, N679K, P681H, N764K, D796Y, Q954H, and N969K were observed among all the three viz., the BA.1.1.529, BA.1.1, and BA.2 lineages. The major mutation del69/70 commonly found in BA.1.1.529 and BA.1.1 was not present in BA.2 as well as not reported in B.1.617.2. The mutations unique to BA.2 were T19I, L24S, P25del, P26del, A27S, V213G, T376A, D405N and R408S. Of these, three specific mutations (T376A, D405N and R408S) are confined to the RBD, which reportedly attributes to ACE2 binding and membrane fusion (40) and transmissibility, which appears to have contributed to the drastic increase in BA.2 cases globally. Owing to these selective mutations, BA.2 is currently suggested to evade neutralizing antibodies induced by vaccination or natural infection (41). Infection with BA.1.1 (R346K) variant appears to result in moderate to severe lung disease like that of the Delta B.1.617.2 variant, and the neutralizing antibodies produced in response to Omicron (R346K) variant infection shows poor neutralizing ability against other co-circulating SARS-CoV-2 variants like Delta B.1.617.2, which necessitates caution as it may lead to increased cases of reinfection. BA.2 mutations T376A, D405N, and R408S may reduce the efficacy of many antibodies. Therefore, it can be speculated that these novel mutations could also increase the disease attributes of BA.2 compared to the earlier lineages.

Concerning the viral transmissibility and pathogenicity; it has been reported that the Omicron variant and its sub-lineage variants harbor an unique D614G mutation in the S protein that also confers enhanced replicative potential and cellular tropism toward the airway epithelial cells as compared to lung cells in experimentally-infected hamsters, likely attributing to its greater degree of transmissibility among humans (42). Another study compared the replication competence and cellular tropism of the wild-type virus and the D614G, Alpha (B.1.1.7), Beta (B.1.351), Delta B.1.617.2 and Omicron (B.1.1.529) variants in *ex vivo* explant cultures of human bronchi and lungs, and showed that the Omicron variant replicates faster than any other variants studied in the bronchi, but less efficiently in the lung parenchyma (43). The lower replication competence of Omicron in the human lungs likely explains its reduced severity that is now being reported in epidemiological studies. This is consistent with our observation where Omicron BA.1.1 and BA.2 had a higher viral load in nasopharyngeal cavity that enhances transmissibility. We also believe that the immune perturbations brought about by the older Delta B.1.617.2 variant in mid-2021 appears to have lowered the ability to induce hypercytokinemia in immune cells involved in the disease, which therefore might have been the rationale behind the less severe COVID-19 brought about by the subsequent Omicron variants. It has been reported that Omicron largely downplays cytokine storm and viral replication (44).

Researchers in England, Scotland, and South Africa have found the risk of admission to hospital to be between 15% and 80% lower with Omicron than the Delta B.1.617.2 variant (45, 46). Though the Omicron variant may cause mild clinical manifestations, the immune escape potential and high transmissibility could likely offset the reduced pathogenesis and disease severity. Furthermore, the determinants of disease severity are multifaceted, and host factors such as comorbidity could likely influence the disease course. We recently reported that age and certain underlying conditions (viz., hypertension, diabetes mellitus and cardiovascular disease) were independently associated with the development of a breakthrough infection (16). As the virus spreads rapidly, the Omicron variant could still progressively overwhelm the healthcare system, and morbidity and mortality rates could surge. Our findings of higher nasopharyngeal viral load among individuals with Omicron variants BA.1.1 and BA.2 infection as compared to those infected with the Delta B.1.617.2 and Omicron BA.1.1.529 variants by ~1.3 fold provide clues to ongoing ebb in vaccine efficacy advocating the need for a vaccine that can confer a broad neutralization potential against all emerging variants rather than merely neutralizing the ancestral wild-type or closely related virus strains. Interestingly, we observed that the BA.2 variant was associated with shorter time-to-death. Many factors are known to impact the prognosis of COVID-19 viz., the age of the individual, the dynamics of antibody responses (47), type/nature of vaccine(s) used (48), interval between the vaccine doses (49, 50), underlying comorbidities and other health issues (51). Given that the neutralizing antibodies induced following vaccination or natural infection decay with time (16) and that the Omicron represents a major variant that dodges the immune system (29), the neutralizing potential of antibodies is almost always weak against BA.2 as compared to its eclectic predecessor variants. Furthermore, the median age of the current cohort is quite high (73 years old) and many of the participants had multiple comorbidities, it is not surprising that BA.2 will have a shorter time-to-death. Given the broader immune escape strategies displayed by Omicron variants (52), improved vaccine preparations based on newer circulating strains should be developed to cater to the needs of the global public.

## Conclusion

Our observation indicates that the low SARS-CoV-2 viral load observed among vaccinated individuals infected with the Delta B.1.617.2 and the Omicron BA.1 variant but not with the BA.1.1 and A.2 variants, hinting that the newer variants likely escape the host's immune responses induced originally by a vaccine that was developed based on an ancestral wild-type virus. Abundant mutations and ongoing emergence of newer variants appear to render viral evasion from neutralizing antibodies in vaccinated individuals. Therefore, the current findings help in understanding the evolutionary imprints of Omicron variants to be able to develop improved antiviral strategies based on the ongoing evolution of SARS-CoV-2.

## Data availability statement

The datasets presented in this study can be found in online repositories. The names of the repository/repositories and accession number(s) can be found in the article/Supplementary material.

## Ethics statement

The studies involving human participants were reviewed and approved by Human Ethics Committee of the Madras Medical College (MMC) (EC No. 03092021). The patients/participants provided their written informed consent to participate in this study.

## Author contributions

STS, YY, NJ, VK, AM, KN, ML, ES, and SS were responsible for conceptualization and data curation. STS, NJ, KH, HT, YZ, GS, MR, AK, RK, VK, AVDM, MK, AM, KN, SP, SS, ML, ES, and SR were responsible for methodology, formal analysis, validation, and visualization. STS, YY, NJ, ML, ES, and SR were responsible for original draft, while all authors have reviewed, edited, and approved the final manuscript.

## Funding

This work was supported by Xiamen University Malaysia Research Funding (XMUMRF), XMUMRF/2018-C2/ILAB/0001 to YY, XMUMRF/2020-C5/ITCM/0003 to HT and XMUMRF/2018-C1/IENG/0005 to YZ, The Swedish Research Council, The Swedish, Physicians against AIDS Research Foundation, The Swedish International Development Cooperation Agency; SIDA SARC, VINNMER for Vinnova, Linköping University Hospital Research Fund, CALF, and The Swedish Society of Medicine (AI52731) to ML, Funding support provided by the Department of Science and Technology-Science and Engineering Research Board, Government of India (CRG/2019/006096) (to ES). The content is solely the responsibility of the authors and does not necessarily represent the views of the official affiliations of the authors.

## Conflict of interest

The authors declare that the research was conducted in the absence of any commercial or financial relationships that could be construed as a potential conflict of interest.

## Publisher's note

All claims expressed in this article are solely those of the authors and do not necessarily represent those of their affiliated organizations, or those of the publisher, the editors and the reviewers. Any product that may be evaluated in this article, or claim that may be made by its manufacturer, is not guaranteed or endorsed by the publisher.
