# Supplementary material for: Low SARS-CoV-2 viral load among vaccinated individuals infected with Delta B.1.617.2 and Omicron BA.1.1.529 but not with Omicron BA.1.1 and BA.2 variants
Source: Front Public Health. 2022 Sep 20;10:1018399. doi: 10.3389/fpubh.2022.1018399 (PMC9540788; doi:10.3389/fpubh.2022.1018399)
Supplement: Supplementary file 1 [file Table_1.pdf]

Supplementary Table 1

Title: Low SARS CoV-2 Viral Load among Vaccinated Individuals Infected with Delta B.1.617.2 and Omicron BA.1.1.529, but not with Omicron BA.1.1 and BA.2 Variants

Accession numbers for the 73 sequences uploaded in GenBank Repository

S.No	Batch ID Generated for Submission	GenBank Accession No
1	SUB11981063 Seq01	OP349592
2	SUB11970912 Seq02	OP295702
3	SUB11970912 Seq03	OP295703
4	SUB11970912 Seq04	OP295704
5	SUB11970912 Seq05	OP295705
6	SUB12002379 Seq06	OP355283
7	SUB11970912 Seq07	OP295706
8	SUB11970912 Seq08	OP295707
9	SUB11970912 Seq09	OP295708
10	SUB11970912 Seq10	OP295709
11	SUB11970912 Seq11	OP295710
12	SUB11970912 Seq12	OP295711
13	SUB12003916 Seq13	OP355302
14	SUB11970912 Seq14	OP295712
15	SUB11970912 Seq15	OP295713
16	SUB12003919 Seq16	OP355303
17	SUB11970912 Seq17	OP295714
18	SUB12003922 Seq18	OP355304
19	SUB12003927 Seq19	OP355305
20	SUB11970912 Seq20	OP295715
21	SUB11970912 Seq21	OP295716
22	SUB11970912 Seq22	OP295717
23	SUB11970912 Seq23	OP295718
24	SUB11970912 Seq24	OP295719
25	SUB11970912 Seq25	OP295720
26	SUB11970912 Seq26	OP295721
27	SUB11970912 Seq27	OP295722
28	SUB11970912 Seq28	OP295723
29	SUB11970912 Seq29	OP295724
30	SUB11970912 Seq30	OP295725
31	SUB11970912 Seq31	OP295726
32	SUB11978946 Seq32	OP320507
33	SUB11970912 Seq33	OP295727
34	SUB11970912 Seq34	OP295728
35	SUB11970912 Seq35	OP295729
36	SUB11970912 Seq36	OP295730
37	SUB11970912 Seq37	OP295731
38	SUB11978946 Seq38	OP320508
39	SUB11970912 Seq39	OP295732

40	SUB11970912 Seq40	OP295733
41	SUB11970912 Seq41	OP295734
42	SUB11970912 Seq42	OP295735
43	SUB11970912 Seq43	OP295736
44	SUB11970912 Seq44	OP295737
45	SUB11970912 Seq45	OP295738
46	SUB11970912 Seq46	OP295739
47	SUB11970912 Seq47	OP295740
48	SUB11978946 Seq48	OP320509
49	SUB11970912 Seq49	OP295741
50	SUB11970912 Seq50	OP295742
51	SUB11970912 Seq51	OP295743
52	SUB11970912 Seq52	OP295744
53	SUB11970912 Seq53	OP295745
54	SUB11970912 Seq54	OP295746
55	SUB11970912 Seq55	OP295747
56	SUB11978946 Seq56	OP320510
57	SUB11978946 Seq57	OP320511
58	SUB11978946 Seq58	OP320512
59	SUB11978946 Seq59	OP320513
60	SUB11970912 Seq60	OP295748
61	SUB11970912 Seq61	OP295749
62	SUB11978946 Seq62	OP320514
63	SUB11970912 Seq63	OP295750
64	SUB11978946 Seq64	OP320515
65	SUB11970912 Seq65	OP295751
66	SUB11970912 Seq66	OP295752
67	SUB11978946 Seq67	OP320516
68	SUB11978946 Seq68	OP320517
69	SUB11970912 Seq69	OP295753
70	SUB11970912 Seq70	OP295754
71	SUB11970912 Seq71	OP295755
72	SUB11970912 Seq72	OP295756
73	SUB11970912 Seq73	OP295757
