# Supplementary material for: Low SARS-CoV-2 viral load among vaccinated individuals infected with Delta B.1.617.2 and Omicron BA.1.1.529 but not with Omicron BA.1.1 and BA.2 variants
Source: Front Public Health. 2022 Sep 20;10:1018399. doi: 10.3389/fpubh.2022.1018399 (PMC9540788; doi:10.3389/fpubh.2022.1018399)
Supplement: Supplementary file 2 [file Table_2.pdf]

Supplementary Table: Sequence database of 73 samples subjected to phylogenetic analysis

S.No	GISAID ACCESSION NO	TN-ID
1	EPI_ISL_11887785	CL -4832
2	EPI_ISL_11887786	CL-4833
3	EPI_ISL_11887788	CL-4837
4	EPI_ISL_11887789	CL-4838
5	EPI_ISL_11887790	CL-4839
6	EPI_ISL_11887791	CL-4856
7	EPI_ISL_11887793	CL-4858
8	EPI_ISL_11887794	CL-4859
9	EPI_ISL_11887800	CL-4884
10	EPI_ISL_11887801	CL-4890
11	EPI_ISL_11887802	CL-4891
12	EPI_ISL_11887804	CL-4893
13	EPI_ISL_11887824	CL-4951
14	EPI_ISL_11887826	CL-4955
15	EPI_ISL_11887828	CL-4957
16	EPI_ISL_11887830	CL-4959
17	EPI_ISL_11887831	CL-4960
18	EPI_ISL_11887833	CL-4962
19	EPI_ISL_11887846	CL-4984
20	EPI_ISL_12223854	CL-4998
21	EPI_ISL_12223856	CL-5004
22	EPI_ISL_12223857	CL-5007
23	EPI_ISL_12223858	CL-5012
24	EPI_ISL_12223859	CL-5022
25	EPI_ISL_12223860	CL-5023
26	EPI_ISL_12223901	CL-5025
27	EPI_ISL_12223867	CL-5036
28	EPI_ISL_12223868	CL-5038
29	EPI_ISL_12223869	CL-5040
30	EPI_ISL_12223871	CL-5043
31	EPI_ISL_12223874	CL-5048
32	EPI_ISL_12223875	CL-5049
33	EPI_ISL_12223878	CL-5052
34	EPI_ISL_12223905	CL-5063
35	EPI_ISL_12223888	CL-5082
36	EPI_ISL_12223889	CL-5083
37	EPI_ISL_12223893	CL-5088
38	EPI_ISL_12223896	CL-5094
39	EPI_ISL_12291174	CL-5100
40	EPI_ISL_12291175	CL-5101
41	EPI_ISL_12291177	CL-5103
42	EPI_ISL_12291178	CL-5104
43	EPI_ISL_12291182	CL-5113
44	EPI_ISL_12291183	CL-5114
45	EPI_ISL_12291184	CL-5115
46	EPI_ISL_12291187	CL-5119
47	EPI_ISL_12291189	CL-5121
48	EPI_ISL_12291190	CL-5122
49	EPI_ISL_12291191	CL-5131
50	EPI_ISL_12291193	CL-5141
51	EPI_ISL_12291200	CL-5154
52	EPI_ISL_12291204	CL-5158
53	EPI_ISL_12291220	CL-5189
54	EPI_ISL_12291225	CL-5194
55	EPI_ISL_12291229	CL-5198
56	EPI_ISL_12291244	CL-5214
57	EPI_ISL_12291245	CL-5217
58	EPI_ISL_12291253	CL-5238
59	EPI_ISL_12291267	CL-5252
60	EPI_ISL_12291284	CL-5268
61	EPI_ISL_12291285	CL-5269
62	EPI_ISL_12291286	CL-5270
63	EPI_ISL_12291287	CL-5271
64	EPI_ISL_12291288	CL-5272
65	EPI_ISL_12291289	CL-5273
66	EPI_ISL_12291292	CL-5276
67	EPI_ISL_12291295	CL-5279
68	EPI_ISL_12291311	CL-5295
69	EPI_ISL_12291315	CL-5299
70	EPI_ISL_12291318	CL-5302
71	EPI_ISL_12291320	CL-5304
72	EPI_ISL_12291321	CL-5305
73	EPI_ISL_12291322	CL-5307
